# A Scopus-based examination of tobacco use publications in Middle Eastern Arab countries during the period 2003–2012

**DOI:** 10.1186/1477-7517-11-14

**Published:** 2014-05-01

**Authors:** Sa'ed H Zyoud, Samah W Al-Jabi, Waleed M Sweileh, Rahmat Awang

**Affiliations:** 1 Poison Control and Drug Information Center (PCDIC), College of Medicine and Health Sciences, An-Najah National University, Nablus 44839, Palestine; 2 Department of Clinical and Community Pharmacy, College of Medicine and Health Sciences, An-Najah National University, Nablus 44839, Palestine; 3 WHO Collaborating Centre for Drug Information, National Poison Centre, Universiti Sains Malaysia (USM), Penang 11800, Malaysia; 4 Department of Pharmacology and Toxicology, College of Medicine and Health Sciences, An-Najah National University, Nablus 44839, Palestine

**Keywords:** Bibliometric, Tobacco smoking, Middle Eastern Arab, Scopus

## Abstract

**Background:**

Tobacco smoking is the main health-care problem in the world. Evaluation of scientific output in the field of tobacco use has been poorly explored in Middle Eastern Arab (MEA) countries to date, and there are few internationally published reports on research activity in tobacco use. The main objectives of this study were to analyse the research output originating from 13 MEA countries on tobacco fields and to examine the authorship pattern and the citations retrieved from the *Scopus* database.

**Methods:**

Data from 1 January 2003 through 31 December 2012 were searched for documents with specific words regarding the tobacco field as 'keywords’ in the title in any 1 of the 13 MEA countries. Research productivity was evaluated based on a methodology developed and used in other bibliometric studies.

**Results:**

Five hundred documents were retrieved from 320 peer-reviewed journals. The greatest amount of research activity was from Egypt (25.4%), followed by the Kingdom of Saudi Arabia (KSA) (23.2%), Lebanon (16.3%), and Jordan (14.8%). The total number of citations for the 560 documents, at the time of data analysis (27 August 2013), was 5,585, with a mean ± SD of 9.95 ± 22.64 and a median (interquartile range) of 3(1–10). The *h*-index of the retrieved documents was 34. This study identified 232 (41.4%) documents from 53 countries in MEA-foreign country collaborations. By region, MEA collaborated most often with countries in the Americas (29.6%), followed by countries in the same MEA region (13.4%), especially KSA and Egypt.

**Conclusions:**

The present data reveal a promising rise and a good start for research productivity in the tobacco field in the Arab world. Research output is low in some countries, which can be improved by investing in more international and national collaborative research projects in the field of tobacco.

## Background

Tobacco use is one of the leading health-care problems in the world. Smoking continues to be the most preventable cause of morbidity and mortality contributing to around half a million deaths every month, a situation that is likely to worsen in the future [1]. Tobacco smoking is on the rise, and as a multi-disciplinary field of study, it has resulted in growing research that takes into account almost all those regions that have experienced the greatest increases in bioscience and health-care science production [2,3]. During the last few years, several studies have measured and analysed the scientific research output from Arab countries [4-9]. In contrast, the evaluation of scientific research output in the field of tobacco use has been poorly explored to date and there are few internationally published reports on research activity regarding tobacco use [10-14]. To the best of our knowledge, there is a lack of data concerning the evaluation of research productivity in the field of tobacco originating from the Arab world [12].

The evaluation of scientific research is an essential task where the purpose of evaluation is to determine, and where possible improve, its productivity. Scientific progress is one of the most important indicators for the community and economic development of different countries [15]. Given that the outcome of scientific activity is only known when the authors communicate their findings to a publication distributed among the scientific community, bibliometric indicators are of great importance [10]. Bibliometric indicators involve the application of statistical methods to scientific publications to obtain the bibliographics for each country. These methods are mainly quantitative and are also used to make pronouncements about qualitative pictures of scientific activities [6,16-18]. Bibliometric analysis is a useful tool to obtain information about the current state of research in particular areas and allows researchers to identify and undertake new lines of research [19,20].

The objectives of this study were to analyse research output from Middle Eastern Arab (MEA) countries in tobacco fields and to examine the authorship pattern and the citations retrieved from the *Scopus* database. A comprehensive online search was performed using SciVerse, Scopus, which is one of the world's largest abstract and citation databases of peer-reviewed literature. Scopus contains 41 million records and covers nearly 18,000 titles from 5,000 publishers worldwide, and provides 100% MEDLINE coverage [21]. This study will lead to a better understanding of the current and future status of research in the tobacco field in the Middle East. Furthermore, the results of this study will help health policy makers and people in academia improve tobacco research in the next decade.

## Methods

This study obtained data from Scopus published from 1 January 2003 through 31 December 2012. The choice of the study duration was based on the assumption that the last decade represents a better picture of the pattern of publications and citations received in a certain field when using bibliometric methods [22-25]. Scientific research productivity in year 2013 was excluded because this year was still open for new journal issues. Therefore, inclusion of the year 2013 would create error and bias.

Elsevier, combining the characteristics of both the Web of Science and PubMed, developed the Scopus database. These characteristics allow for enhanced utility for academic needs, medical literature research, and citation analysis. Scopus offers a basic search, a quick search, an author search, an advanced search, and a source search. In the basic search, the results for the chosen keywords can be limited by the date of publication, by subject area, and by document type, whereas the author search is based only on author names [26]. The search results in Scopus can be displayed as a list of 20–200 items per page, and documents can be saved to a list and/or exported. The results can be refined by document type, source title, author name, year of publication, and/or subject area, and a new search can be initiated within the results [26].

The keywords entered into Scopus, in order to accomplish the objectives of this study, were 'smoking’, 'tobacco’ and 'nicotine’, 'snuff’, 'secondhand’, 'smokeless’, 'smoker’, 'cigar’, 'cigarette’, 'smoke’, 'antismoking’, 'nonsmoking’, 'waterpipe’, 'hookah’, 'hubble-bubble’, 'narghile’, 'argila’, and 'shisha’ as 'Article Title’. Then, all 13 Arab countries in the Middle East were entered as country affiliation [i.e. Egypt, Syrian Arab Republic (SAR), Lebanon, Jordan, Iraq, Kingdom of Saudi Arabia (KSA), Kuwait, Bahrain, State of Palestine, United Arab Emirates (UAE), Yemen, Oman, and Qatar]. The subject areas selected for this research were health sciences, life sciences, social sciences, and physical sciences. The resulting search was as follows: your query: (AFFILCOUNTRY (egypt) OR AFFILCOUNTRY (palestine) OR AFFILCOUNTRY (jordan) OR AFFILCOUNTRY (syrian) OR AFFILCOUNTRY (lebanon) OR AFFILCOUNTRY (iraq) OR AFFILCOUNTRY (kuwait) OR AFFILCOUNTRY (qatar) OR AFFILCOUNTRY (united arab) OR AFFILCOUNTRY (bahrain) OR AFFILCOUNTRY (oman) OR AFFILCOUNTRY (yemen) OR AFFILCOUNTRY (saudi) AND TITLE (smoking) OR TITLE (tobacco) OR TITLE (nicotine) ORTITLE (snuff) OR TITLE (secondhand) OR TITLE (smokeless) OR TITLE (smoker) OR TITLE (smoker) OR TITLE (cigar) OR TITLE (cigarette) OR TITLE (smoke) OR TITLE (antismoking) OR TITLE (nonsmoking) OR TITLE (waterpipe) ORTITLE (hookah) OR TITLE (hubble-bubble) OR TITLE (narghile) OR TITLE (argila) OR TITLE (shisha) AND NOT TITLE (flue-cured) AND NOT TITLE (fish) AND NOT TITLE (hornworm) AND NOT TITLE (wood) AND NOT TITLE (plant) AND NOT TITLE (fire) AND NOT TITLE (insect)) AND PUBYEAR > 2002 AND PUBYEAR < 2013. Keywords usedin this study were selected on the basis of another previous similar study [13]. We excluded documents that contained the following keywords: 'flue-cured’, 'fish’, 'hornworm’, 'wood’, 'plant’, 'fire’, and 'insect’. We also excluded those documents in which the primary focus was not a dimension of tobacco research, was not a primary variable of interest, or had as its focus the smoking of substances other than tobacco, such as marijuana.

The collated data were used to generate the following information: (a) total and trends of contributions in tobacco research between 2003 and 2012, (b) MEA countries research productivity, (c) collaboration patterns, (d) journals in which MEA researchers publish, and (e) the citations received by the publications.

### Ethical approval

The Institutional Review Board (IRB) at An-Najah National University does not require submission of an IRB application for a bibliometric study. The IRB confirmed that there is no risk for human subjects in this type of research since the data are based on published literature and, as secondary data, did not involve any interactions with human subjects.

### Statistical analysis

Data from Scopus were exported to Excel and then to the Statistical Package for Social Sciences (SPSS; SPSS Inc., Chicago, IL, USA) program version 15 for analysis. Continuous data are presented as mean ± standard deviation (SD), and categorical data are expressed as numbers with percentages. Variables that are not normally distributed are expressed as a median (Q1–Q3: interquartile range). The measurements of bibliometric analysis (e.g. countries, cited articles, institutions) were converted to the rank order using the standard competition ranking (SCR). We took in our consideration only the ten top-ranked. If the measurements of bibliometric analysis have the same ranking number, then a gap is left in the following ranking numbers. The *h*-index for data collected from Scopus is presented. The *h*-index is a country's number of articles (*h*) that have received at least *h* citations. It quantifies both country scientific productivity and scientific impact, and it is also applicable to scientists, journals, etc. [27]. That is to say, a country with an *h*-index of 10 has published 10 documents, each have attracted at least 10 citations. Documents with fewer than 10 citations are not calculated by the index. The *h*-index was originally developed as a way of qualifying research performance [28]. Two common performance indicators were considered for the top-ten ranked journals using data from the most recent year available [29]. First, the journal impact factor (IF) was evaluated using the Journal Citation Report (JCR; Web of Knowledge) 2012 science edition by Thomson Reuters (New York, NY, USA).The second measure of journal performance used in the current study was the *SCImago Journal Rank* (SJR) indicator. A detailed explanation on how the SJR calculation is made can be found on the SCImago website [30].

## Results

Using the methodology stated above, 560 documents were retrieved comprising 475 (84.8%) original journal articles, 30 (5.4%) review articles, 30 (5.4%) letters to the editor, and 32 (5.7%) other types of publications, with an average of 56 documents per year from 13 MEA countries. The annual number of documents published in the past decade: 2003–2012, indicates that tobacco research productivity during the past decade was low in the first few years but showed an obvious doubling after 2008. The quantity of publications has increased by around threefold from 2003 to 2012.

The retrieved documents were published in 320 peer-reviewed journals. Table 1 shows a list ranking the top 10 journals in which tobacco-related articles from 13 MEA authors were published. Twenty-nine documents (5.2%) were published in *Eastern Mediterranean Health Journal* whereas 24 (4.3%) were published in *Saudi Medical Journal*, 15 (2.7%) were published in *Nicotine and Tobacco Research*, and 12 (2.1%) were published in *Tobacco Control*. Four journals in the top 10 ranking journals had SJR >1. Furthermore, one journal in the top 10 ranking journals had no official IF and was not listed in the JCR 2012.

**Table 1:** The top 10 journals from 320 journals which published tobacco-related articles from 13 MEA countries

**SCR**^**a**^	**Journal**	**Frequency (%)**	**SJR**	**IF (2012)**^**b**^
1st	*Eastern Mediterranean Health Journal*	29 (5.2)	0.27	NA
2nd	*Saudi Medical Journal*	24 (4.3)	0.23	0.619
3rd	*Nicotine and Tobacco Research*	15 (2.7)	1.23	2.477
4th	*Tobacco Control*	12 (2.1)	1.62	4.111
5th	*BMC Public Health*	8 (1.4)	0.98	2.076
5th	*International Journal of Tuberculosis and Lung Disease*	8 (1.4)	1.34	2.61
5th	*Food and Chemical Toxicology*	8 (1.4)	0.99	3.01
5th	*Annals of Saudi Medicine*	8 (1.4)	0.38	1.103
9th	*Asian Pacific Journal of Cancer Prevention*	7 (1.3)	0.31	1.271
10th	*Preventive Medicine*	6 (1.1)	1.62	3.496
10th	*Annals of Thoracic Medicine*	6 (1.1)	0.38	1.123

*SCR* standard competition ranking, *SJR* SCImago Journal Rank, *NA* not available, *IF* impact factor. ^a^Equal journal has the same ranking number, and then a gap is left in the ranking numbers; ^b^the impact factor was reported according to Institute for Scientific Information (ISI) Journal Citation Report (JCR) 2012.

When the data were analysed by country, the greatest amount of research activity was from Egypt (25.4%), followed by KSA (23.2%), Lebanon (16.3%), and Jordan (14.8%) (Table 2). The total number of citations for the 560 documents, at the time of data analysis (27 August 2013), was 5,585 with a mean ± SD of 9.95 ± 22.64 and a median (interquartile range) of 3(1–10). Of the 560 documents considered for the *h*-index, 34 were cited at least 34 times. The highest *h*-index was 23 for Lebanon, followed by 21 for SAR, 16 for Egypt, and the lowest *h*-index was 1 for Palestine. Furthermore, the highest percentage of documents in collaboration with international authors from the total number of documents for each country was 92.7% for SAR followed by 84.6% for Yemen (Table 2). Table 3 presents a list of the 10 most cited documents originating from MEA countries.

**Table 2:** Bibliometric analysis of the 560 documents by country

**Country**	**Number of documents *N*** **= 560 (%)**^**a**^	***h-*** **index**	**Number (%)**^**b**^** of documents with international authors**
Egypt	142 (25.4)	16	60 (42.3)
Kingdom of Saudi Arabia	130 (23.2)	15	57 (43.8)
Lebanon	91 (16.3)	23	46 (50.5)
Jordan	83 (14.8)	13	40 (48.2)
Syrian Arab Republic	55 (9.8)	21	51 (92.7)
Kuwait	41(7.3)	12	21 (51.2)
United Arab Emirates	28 (5.0)	2	17 (60.7)
Bahrain	16 (2.9)	4	6 (37.5)
Iraq	14 (2.5)	3	7 (50.0)
Yemen	13 (2.3)	6	11 (84.6)
Qatar	11(2.0)	4	8 (72.7)
Oman	9 (1.6)	4	5 (55.6)
Palestine	2 (0.4)	1	1 (50.0)

^a^Total exceeds 100% because data are overlapping due to multiple collaborations; ^b^percentage of documents with international authors from the total number of documents for each country.

**Table 3:** The top 10 cited tobacco-related articles from the 13 MEA countries in Scopus

**SCR**	**Authors with year of publication**	**Title**	**Journal name**	**Times cited**
1st	Teo et al. 2006	Tobacco use and risk of myocardial infarction in 52 countries in the INTERHEART study: a case-control study	*Lancet*	337
2nd	Maziak et al. 2004	Tobacco smoking using a waterpipe: a re-emerging strain in a global epidemic	*Tobacco Control*	179
3rd	Shihadeh 2003	Investigation of mainstream smoke aerosol of the argileh water pipe	*Food and Chemical Toxicology*	155
4th	Degenhardt et al. 2008	Toward a global view of alcohol, tobacco, cannabis, and cocaine use: findings from the WHO world mental health surveys	*PLoS Medicine*	150
5th	Shihadeh and Saleh 2005	Polycyclic aromatic hydrocarbons, carbon monoxide, “tar”, and nicotine in the mainstream smoke aerosolof the narghile water pipe	*Food and Chemical Toxicology*	134
6th	Tamim et al. 2003	Tobacco use by university students, Lebanon, 2001	*Addiction*	100
7th	Eissenberg et al. 2008	Waterpipe tobacco smoking on a U.S. college campus: prevalence and correlates	*Journal of Adolescent Health*	80
8th	Smith-Simone et al. 2008	Waterpipe tobacco smoking: knowledge, attitudes, beliefs, and behavior in two U.S. samples	*Nicotine and Tobacco Research*	77
9th	Akl et al. 2010	The effects of waterpipe tobacco smoking on health outcomes: a systematic review	*International Journal of Epidemiology*	76
10th	Maziak et al. 2004	Prevalence and characteristics of narghile smoking among university students in Syria	*International Journal of Tuberculosis and Lung Disease*	73

*SCR* standard competition ranking.

The study identified 232 (41.4%) documents from 53 countries in MEA-foreign country collaborations (Table 4). MEA actively collaborated with authors from the USA (*n* = 150, the highest number recorded), followed by Canada (*n* = 26), and Germany (*n* = 22); (Table 4). By region, MEA countries collaborated most with countries in the Americas (29.6%), followed by countries in the same MEA region (13.4%), especially KSA and Egypt (Table 4).

**Table 4:** Collaborations between the 13 MEA countries and foreign countries in tobacco-related publications

**Collaborating countries**^**a**^	**Number of documents**	**Collaborating countries**	**Number of documents**
MEA-MEA	75 (13.4%)	MEA-Europe	68 (12.1%)^b^
Saudi Arabia	25	Germany	22
Egypt	23	UK	19
Jordan	17	France	9
Syrian Arab Republic	14	Netherlands	8
Lebanon	13	Spain	7
United Arab Emirates	10	Italy	6
Bahrain	6	Belgium	4
Kuwait	5	Denmark	4
Qatar	5	Sweden	4
Oman	5	Greece	4
Iraq	4	Ireland	4
Yemen	3	Finland	3
Palestine	1	Hungary	3
MEA-other Middle East, Africa	18 (3.2%)^b^	Switzerland	3
Turkey	5	Austria	3
Nigeria	5	Romania	3
Morocco	4	Ukraine	2
Tunisia	4	Poland	2
South Africa	4	Estonia	1
Israel	3	Russian Federation	1
Uganda	3	Latvia	1
Algeria	2	Serbia	1
Libyan Arab Jamahiriya	1	Lithuania	1
Kenya	1	Czech Republic	1
MEA-Americas	166 (29.6%)^b^	Bulgaria	1
USA	150	MEA-Asia-Pacific	36 (6.4%)^b^
Canada	26	India	11
Brazil	4	Japan	11
Mexico	4	Australia	7
Colombia	3	China	7
Argentina	2	Pakistan	7
MEA-Southeast Asia	5 (0.9%)^b^	New Zealand	3
Malaysia	5	Taiwan	2
MEA-other (Norway)	1 (0.2%)^b^	Hong Kong	1
		South Korea	1
		Nepal	1

^a^The study identified 232 (41.4%) documents with 53 countries in MEA-foreign country collaborations; ^b^total exceeds 41.4% as data are overlapping due to multi-country collaboration.

Table 5 shows the top 10 productive institutions in tobacco research from MEA affiliations or collaborated with MEA authors. The most productive institutions were American University of Beirut (12.0% of total publications), King Saud University (10.9%), Jordan University of Science and Technology (8.0%), and University of Kuwait (7.7%). Table 6 shows the top 10 most prolific authors in the field of tobacco research from the 13 MEA countries with their affiliations and publication patterns. Each of those authors has published at least eight articles during the period of the study.

**Table 5:** Top 10 productive institutions from MEA or collaborated with the 13 MEA affiliations during the study period

**SCR**	**Institutions**	**Number of documents (%)**
1st	American University of Beirut	67 (12.0)
2nd	King Saud University	61 (10.9)
3rd	Jordan University of Science and Technology	45 (8.0)
4th	University of Kuwait	43 (7.7)
5th	Virginia Commonwealth University	42 (7.5)
6th	Syrian Center for Tobacco Studies	38 (6.8)
7th	Cairo University	34 (6.1)
8th	Ain Shams University	34 (6.1)
9th	University of Memphis	29 (5.2)
10th	King Abdulaziz University	24 (4.3)

*SCR* standard competition ranking.

**Table 6:** Top 10 prolific authors in the field of tobacco from the 13 MEA countries

**SCR**^**a**^	**Author**	**Number (%)**^**b**^** of tobacco publications**	**Affiliation**
1st	Eissenberg, T.	37 (6.6)	Virginia Commonwealth University, Institute for Drug and Alcohol Studies, Richmond, USA
2nd	Maziak, W.	32 (5.7)	Florida International University, Robert Stempel College of Public Health and Social Work, Miami, USA
3rd	Ward, K.D.	25 (4.5)	University Memphis, School of Public Health, Memphis, USA
4th	Shihadeh, A.	23 (4.1)	American University of Beirut, Department of Mechanical Engineering, Beirut, Lebanon
4th	Alzoubi, K.H.	16 (2.9)	Jordan University of Science and Technology, Department of Clinical Pharmacy, Irbid, Jordan
6th	Rastam, S.	15 (2.7)	University of Aleppo, School of Medicine, Aleppo, Syrian Arab Republic
7th	Alkadhi, K.A.	11 (2.0)	University of Houston, Department of Pharmacological and Pharmaceutical Sciences, Houston, USA
8th	Asfar, T.	9 (1.6)	University of Miami Leonard M. Miller School of Medicine, Department of Epidemiology and Public Health, Miami, USA
8th	Aleisa, A.M.	9 (1.6)	King Saud University College of Pharmacy, Department of Pharmacology, Riyadh, Saudi Arabia
9th	El-Mas, M.M.	8 (1.4)	Alexandria University Faculty of Pharmacy, Department of Pharmacology and Toxicology, Alexandria, Egypt
9th	Warren, C.W.	8 (1.4)	Centers for Disease Control and Prevention, Office on Smoking and Health, Atlanta, USA
9th	Tamim, H.	8 (1.4)	York University, Faculty of Health Sciences, Toronto, Canada
9th	Jones, N.R.	8 (1.4)	Penn State College of Medicine, Department of Pharmacology, Hershey, USA
9th	Nakkash, R.	8 (1.4)	American University of Beirut, Department of Health Promotion and Community Health, Beirut, Lebanon
9th	El-Gowilly, S.M.	8 (1.4)	Alexandria University Faculty of Pharmacy, Department of Pharmacology and Toxicology, Alexandria, Egypt

*SCR* standard competition ranking. ^a^Equal authors have the same ranking number, and then a gap is left in the ranking numbers; ^b^percentage of publications for each author by the total number of documents.

## Discussion

Reducing tobacco-related death and disease in the Arab world requires an understanding of how these various countries have progressed in scientific tobacco research. Such understanding is instrumental for the development of an effective plan to respondto the issue based on research progress and garner public and political support for it [31]. This study was limited to 560 documents extracted from Scopus, bearing affiliation addresses from MEA countries and, therefore, cannot be generalised to the tobacco literature covered by other databases such as Google Scholar. However, the study does give a clear picture about the characteristics of the documents from MEA countries published in foreign channels, especially those indexed by Scopus. Although the number of citations for each publication might differ from one search engine to another, the Scopus search engine remains one of the best available tools for analysing and tracking citations and comparing citations to different research groups and different institutions [2]. Studies that compared PubMed, Scopus, Web of Knowledge, and Google Scholar found that PubMed remains an important resource for clinicians and researchers, while Scopus covers a wider journal range and offers the capability for citation analysis [2,26,32]. On the other hand, there are various reasons for using Scopus database exclusively in the current study. Firstly, it has been shown that Scopus can be used as the sole data source for bibliometric-based research in certain fields [29,33], including tobacco use [13]. Secondly, Scopus has a relatively large database of source journal and includes a more expanded spectrum of journals than PubMed and Web of Science [26]. Thirdly, although other Internet-wide search engines such as *Google Scholar* may be useful for identifying 'grey literature’ (i.e. older, non-listed journals), this method is known to be very time-consuming when compared to some of the more scientifically orientated databases [33]. It is obvious that Google Scholar makes citations only to articles that were electronically accessible. The use of Google Scholar to determine citations for a particular article is disappointing, because of its inadequacies, its inclusion of non-scholarly citations, and the fact that much information about its content coverage remains unknown [26,34].

In the present study, bibliometric indicators were used to describe scientific activity in the field of tobacco usage in 13 MEA countries during the last decade. To the best of the authors' knowledge, this is the first article to analyse the quantity and quality of tobacco-based research from the Arab world. Research indicators showed that research activity in this field was neglected in most MEA countries. This paper also adds to the emerging bibliometric literature within tobacco research [11,13,14]. Fight against tobacco smoking and search for effective tobacco cessation methods have been largely enhanced by the scientific work of the Cochrane Tobacco Addiction Group (CTAG), whose goal is to produce up-to-date and reliable systematic reviews of interventions for the cessation and prevention of tobacco use. By September 2013, the CTAG produced 68 full reviews about tobacco cessation in high-impact journals [35]. Our study about the bibliometrics of tobacco use would uniquely add up to the CTAG efforts in combating tobacco control at the regional and the global level.

The total publications found in Scopus between 2003 and 2012 showed a yearly increase. Most countries experienced increases in the absolute number of documents produced in the field of tobacco over time. Tobacco research productivity has followed the general explosion in scientific productivity observed in the last decade and especially in recent years [8,36,37]. As can be seen in our study, the behaviour of every country was different. Our study showed that there were some countries, such as Egypt and KSA, where the total tobacco research productivity during this 10-year period was clearly higher than the remaining countries. This activity depends on population, socio-economic,or overall scientific activity of the country [37]. Socio-economic aspects can also influence smoking rates within a different population [38,39]. Several studies demonstrated that socio-economic factors, especially educational level, annual household income, and occupational class, have a strong influence on smoking behaviour [38,39]. Therefore, it would have been more interesting to know how the growth of tobacco research in these countries differed in quality rather than in quantity, as shown by the *h*-index for each country. The preparation of quality research documents requires significant effort and time. Publishing high-quality research allows established researchers to be able to obtain further funding to support collaborative research and for young researchers to be more competitive in career advancement [40].

The number of articles with international collaboration was high. Besides the USA, countries from the MEA region with low scientific tobacco research would benefit from more collaboration with the European region because international collaboration articles with high citations per documents have been co-authored with researchers from these countries. Moreover, MEA authors mainly collaborated with authors from the USA, UK, Germany, Canada, India, and Japan. This may be because most MEA academics graduated from or were trained in these countries. Investigators who are open to collaborations and those who seem to adequately manage their collaborations produce a superior product that results in a higher impact and higher citation rates [41]. The factors in favour of increasing collaborations internationally cannot be ignored; these are the results of easier access to public financing, opportunities to attain higher productivity, and aspirations for greater prestige and visibility resulting from collaboration with renowned research groups [3,18,42,43].

In addition to these advantages of collaboration, follow-up research expertise of other countries, developed or developing, is another key factor for facilitating applicable and translatable research in countries that historically lack it. de Granda-Orive and colleagues [3] examined scientific collaboration in the published literature on smoking over a5-year period. They found that the UK published the highest number of documents with international collaboration, followed by the USA and Germany, whereas the USA published the highest number of articles with inter-institutional collaboration, followed by the UK and France. Articles resulting from inter-institutional collaborations received a higher number of citations than those with no collaborations [3]. Furthermore, Kusma and colleagues [44] found that Canada and the USA are the leading cooperating countries. This was followed by the cooperation between Australia and the USA and the UK and the USA [44].

Institutions of higher learning, both public and private, dominated the top 10 productive institutions for research publications in the field of tobacco, indicating that institutions of higher learning were actively researching in the tobacco field and were successful in making their contributions visible through Scopus-indexed journals. This may be attributed to the emphasis by universities for academics to publish in journals indexed by the Scopus databases. Information about trends and productivity reveals the intellectual output of tobacco works published in Scopus and is useful to university administrators when evaluating yearly performance of university faculty in light of university ranking among various universities [45]. This study reports on the most prolific authors from MEA countries with their affiliations and publication patterns, indicating their active roles as writers in the field of tobacco. In some MEA universities, promotional criteria required academics to show their active involvement in research, as reflected by the ranking of the most prolific authors in a certain field. Often, the Division of Research and Innovation will be asked by university administrators to provide such evidence, and the analysis of the names of productive authors becomes necessary.

To the best of our knowledge, this study is the first of its kind to obtain initial data regarding the publication and citation productivity of MEA countries in the tobacco field in the Scopus database, a database that is being used to evaluate the performance of institutes and their members. This study is not without limitations, most of which are the same as those of studies performed in other biomedical fields [22-25]. First of all, we used Scopus criteria for including tobacco-related keywords in our study. Articles published in non-Scopus-cited journals were not included, although they might contribute to scientific productivity. Another limitation is that some international journals do not recognise countries like Palestine as a separate country and publications from Palestine may be affiliated with Israel as a country. Therefore, some publications from Palestine might be missing from our analysis. Another limitation is that some articles did not point out tobacco and related terms in article titles; however, these terms were mentioned throughout the text. Therefore, it is possible that the number of publications analysed in this study did not exactly represent all tobacco-based research activity. Furthermore, some conference abstracts may be published by certain journals which may then be published in the same or different journals in a different year as original journal articles. Finally, it should be noted that research output for certain authors or institutions could have been under-estimated because of writing their English names differently in different articles. Therefore, such authors might have two or more author or institute profiles in Scopus because their names were written differently in different articles.

## Conclusions

The present data reveal a promising rise and a good start for research activity in the tobacco field from the Arab world. The quantity of tobacco-based research originating from MEA countries was low for some countries. More effort is needed to bridge the gap in tobacco-based research and to promote better evaluation of tobacco use or control services in MEA countries. The main goal of our study was to direct attention to and to open doors for a scientific discussion among professionals and academics about tobacco research. Academic institutions in the Middle East are advised to initiate tobacco cessation-specialised programmes and to strengthen research collaboration with international researchers and institutions in which tobacco research has evolved. For future studies in this direction, it is recommended that similar quantitative and qualitative research analyses for other disciplines, based on the same methodology, should be compiled for MEA countries.

## Abbreviations

CTAG: Cochrane Tobacco Addiction Group; IFs: impact factors; IRB: Institutional Review Board; ISI: Institute for Scientific Information; JCR: Journal Citation Report; MEA: Middle Eastern Arab; Q1–Q3: lower quartile upper quartile; SAR: Syrian Arab Republic; SCR: standard competition ranking; SD: standard deviation; SJR: SCImago Journal Rank; SPSS: Statistical Package for Social Sciences; UAE: United Arab Emirates.

## Competing interests

The authors declare that they have no competing interests.

## Authors' contributions

SZ conceived of and designed the study, organised and supervised the data collection, provided analysis and interpretation, and wrote the manuscript. SA and WS participated in the study design and provided critical revision of the manuscript for important intellectual content. RA was involved in the conception and editing of the manuscript.All authors were involved in drafting the article, and all authors approved the final version to be submitted for publication.

## Authors' information

Dr. SZ is an assistant professor of clinical toxicology/pharmacy and the head of a research group (WS and SA) in the field of clinical toxicology, clinical pharmacology/pharmacy, social pharmacy, pharmacoepidemiology and drug safety, and bibliometric analysis. SZ and the research group have published many articles in leading international and high-reputation journals. The research group has also supervised many students in the fields of nursing, public health, and pharmacy.
